# Draft genome sequences of five multidrug-resistant *Escherichia coli* strains isolated from vegetable samples in Bangladesh

**DOI:** 10.1128/mra.00982-23

**Published:** 2023-12-14

**Authors:** Md. Saiful Islam, Pritom Kumar Pramanik, Md. Liton Rana, Srinivasan Ramasamy, Pepijn Schreinemachers, Ricardo Oliva, Md. Tanvir Rahman

**Affiliations:** 1 Department of Microbiology and Hygiene, Faculty of Veterinary Science, Bangladesh Agricultural University, Mymensingh, Bangladesh; 2 Department of Animal Sciences, University of California—Davis, Davis, California, USA; 3 World Vegetable Center, Tainan, Taiwan; 4 World Vegetable Center, Bangkok, Thailand; University of Maryland School of Medicine, Baltimore, Maryland, USA

**Keywords:** vegetables, gardening systems, *Escherichia coli*, whole-genome sequencing, MDR, antibiotic resistance genes, virulence factor genes, public health, Bangladesh

## Abstract

Reports indicate that vegetables are becoming a source of multidrug-resistant (MDR) bacteria, including *Escherichia coli*. Here, we present genome sequences of five MDR *E. coli* strains to assist future genomic analysis of this bacterium. These *E. coli* strains were isolated from vegetable samples of different gardening systems in Dhaka, Bangladesh.

## ANNOUNCEMENT

The overuse of antibiotics has led to the development of antibiotic resistance, giving rise to a wide range of multidrug-resistant (MDR) strains that present a substantial global health threat (1, 2). The versatile and adaptable capabilities of *Escherichia coli* make it very commonly found in food, water, and soil (3). The presence of MDR *Escherichia coli* in vegetables poses a public health issue by transferring to the human population via the food supply chain.

Between September 2022 and March 2023, fresh vegetable samples (Table 1) were collected from different gardening systems from Dhaka (23.8105°N, 90.3372°E) district of Bangladesh and transported to the laboratory (24.7245°N, 90.4372°E). The samples were processed following the procedures of the previous study (4). Briefly, vegetable samples were aseptically chopped, weighed (50 g), and placed into a sterile polyethene stomacher bag containing 200 mL of buffered peptone water and macerated for 5 min at 230 rpm in a Stomacher 400 circulator (Seward Ltd., London, UK). The processed samples were then incubated at 37°C for 24 h, spread on eosin methylene blue agar plates, and incubated at 37°C overnight. The resulting colonies were subjected to Gram staining and biochemical tests (indole, methyl red, Voges-Proskauer, citrate utilization, and sugar fermentation tests) to isolate *E. coli* (5). *E. coli* identification was then performed using matrix-assisted laser desorption ionization time-of-flight mass spectrometry (6). For this study, five MDR *E. coli* strains (MTR_DSS_V097, MTR_DSS_V098, MTR_DNS_V20, MTR_DSR_V504, and MTR_DNS_V06) were chosen and incubated in nutrient broth (HiMedia, India) at 37°C overnight. DNA was subsequently extracted from the cultured broth using a DNeasy Blood and Tissue Kit (QIAGEN, Hilden, Germany). The DNA library was prepared using the Nextera DNA Flex Library Prep Kit (Illumina, San Diego, CA, USA). Genome sequencing was carried out on the Illumina NextSeq2000 platform, which generated paired-end reads with a length of 2 × 150 bp. The genome assembly was conducted using Unicycler v.0.4.9 (7), following a preliminary step of trimming the raw paired-end reads (Table 1) with Trimmomatic v.0.39 (8) (leading: 20, sliding window: 4:20:20, trailing: 20, and minlen = 36) to remove Illumina adapters, known Illumina artifacts, and phiX reads from the data set. Quality assessment was performed using FastQC v.0.11.7 (9). The annotation of the genome was done using PGAP v.6.6 (10). In our assembled genome, sequence types were predicted by MLST v.2.0 (11), pathogenicity index by PathogenFinder v.1.1 (12), CRISPR arrays and prophages by CRISPRimmunity (13), plasmids by PlasmidFinder v.2.1 (14), antibiotic resistance genes (ARGs) by CARD v.3.2.4 with RGI main [the ARGs selection criteria were set to perfect (100% identity) and strict (>95% identity) hits only to the curated reference sequences in the CARD databases] (15) and ResFinder v.4.1 (16); virulence factor genes (VFGs) by VFDB with VFanalyzer (17) and VirulenceFinder v.2.0 (18); siderophores by antiSMASH v.7.0 (19); bacteriocins by BAGEL suite v.4.0 (20); and metabolic functional features by RAST v.2.0 (21). Default parameters were used for all software unless otherwise specified.

**TABLE 1:** Genomic features of the sequenced five multidrug-resistant *Escherichia coli* strains isolated from different vegetable samples from urban and peri-urban gardening systems in Dhaka, Bangladesh

	MTR_DSS_V097	MTR_DSS_V098	MTR_DNS_V20	MTR_DSR_V504	MTR_DNS_V06
**Samples (scientific name)**	Red amaranth (*Amaranthus cruentus*)	Radish leaves (*Raphanus sativus*)	Green chilies (*Capsicum frutescens*)	Tomato (*Solanum lycopersicum*)	Malabar spinach (*Basella alba*)
BioProject accession no.	PRJNA1019895				
BioSample accession no.	SAMN37503130	SAMN37503131	SAMN37503129	SAMN37503132	SAMN37503128
SRA accession no.	SRR26156588	SRR26156587	SRR26156586	SRR26156585	SRR26156584
GenBank accession no.	JAVTVL000000000	JAVTVM000000000	JAVTVN000000000	JAVTVO000000000	JAVTVP000000000
Total length (bp)	4,745,385	4,773,716	4,331,091	4,778,128	4,896,184
Coverage (*x*)	55.0404	73.5677	80.033	60.0396	69.8073
No. of contigs	105	142	125	134	128
No. of reads	1,043,863	1,454,216	1,424,402	1,146,569	1,461,125
Contig L50	7	14	19	11	11
Contig N50 (bp)	205,460	147,301	77,403	152,699	137,565
GC content (%)	50.67	50.69	50.93	50.69	50.80
Genes (total)	4,630	4,710	4,225	4,718	4,862
Coding Sequences (CDSs) (total)	4,548	4,627	4,145	4,627	4,773
Genes (coding)	4,447	4,476	4,030	4,474	4,589
CDSs (with protein)	4,447	4,476	4,030	4,474	4,589
No. of RNA genes (tRNAs, rRNAs, and ncRNAs)	82 (71, 2, and 9)	83 (72, 2, and 9)	80 (69, 2, and 9)	91 (78, 2, and 11)	89 (77, 3, and 9)
Pseudo genes (total)	101	151	115	153	184
Sequence types (STs)	441	2	1162	2	398
Pathogenicity index	0.944	0.932	0.945	0.933	0.937
No. of Clustered Regularly Interspaced Short Palindromic Repeats (CRISPR) arrays (no. of genes) (list of genes)	2 (10) (*csa3*, *cas2*, *cas1*, *cas6e*, *cas5*, *cas7*, *cse2gr11*, *cas8e*, *cas3*, and *WYL*)	2 (10) (*WYL*, *cas3*, *cas8e*, *cse2gr11*, *cas7*, *cas5*, *cas6e*, *csa3*, *cas2*, and *cas1*)	3 (8) (*cas5*, *cas6e*, *cas1*, *cas2*, *csa3*, *cas8e*, *cas3*, and *WYL*)	2 (10) (*WYL*, *cas3*, *cas8e*, *cse2gr11*, *cas7*, *cas5*, *cas6e*, *cas1*, *cas2*, and *csa3*)	3 (12) (*cas5*, *cas6e*, *cas1*, *cas2*, *csa3*, *DEDDh*, *TnsC*, *WYL*, *cas3*, *cas8e*, *cse2gr11*, and *cas7*]
No. of prophages	10	16	12	18	14
Plasmids	1 [IncFIB(AP001918)]	3 [IncFIA, IncFIB(AP001918), IncX1]	0	3 [IncFIA, IncFIB(AP001918), IncX1]	2 [IncFIB(AP001918), IncY]
Predicted Antibiotic Resistance Genes (ARGs) in CARD	53	68	55	68	56
Predicted Virulence Factor Genes (VFGs) in VFDB	64	62	32	59	82
Predicted siderophores	Saccharide and enterobactin	Saccharide and enterobactin	Saccharide and enterobactin	Saccharide and enterobactin	Saccharide and enterobactin
Predicted bacteriocins	Bottromycin	Sactipeptides nd bottromycin	Bottromycin	Bottromycin	Bottromycin, 77.3;lin_M18
No. of subsystems (coverage, genes)	378 (33%, 2,112)	374 (32%, 2,079)	365 (34%, 1,981)	374 (32%, 2,082)	79 (31%, 2,086)

Genomic characteristics of all the five *E. coli* strains are presented in Table 1. The *E. coli* strains MTR_DSS_V097, MTR_DSS_V098, MTR_DNS_V20, MTR_DSR_V504, and MTR_DNS_V06 contained 53, 68, 55, 68, and 56 predicted ARGs, respectively, and 64, 62, 32, 59, and 82 VFGs, respectively. Plasmids, such as IncFIA, IncFIB(AP001918), IncX1, and IncY were harbored in these strains. Four different sequence types (ST2, ST398, ST441, and ST1162) were identified in these strains, and the pathogenicity indices varied from 93% to 95%. The number of CRISPR arrays varied from two to three with 8–12 genes; the prophage numbers ranged between 10 and 18. Two types of siderophores and three types of bacteriocins were identified in the sequenced genomes. Moreover, 378, 374, 365, 359, and 79 subsystems were identified in the *E. coli* strains MTR_DSS_V097, MTR_DSS_V098, MTR_DNS_V20, MTR_DSR_V504, and MTR_DNS_V06, respectively.

## Data Availability

See Table 1 for a list of accession numbers reported on.
